# Insights into vehicle conflicts based on traffic flow dynamics

**DOI:** 10.1038/s41598-023-50017-3

**Published:** 2024-01-17

**Authors:** Shengxuan Ding, Mohamed Abdel-Aty, Zijin Wang, Dongdong Wang

**Affiliations:** Department of Civil, Environmental and Construction Engineering, University of Central Florida, Orlando, FL 32816 USA

**Keywords:** Civil engineering, Risk factors

## Abstract

The utilization of traffic conflict indicators is crucial for assessing traffic safety, especially when the crash data is unavailable. To identify traffic conflicts based on traffic flow characteristics across various traffic states, we propose a framework that utilizes unsupervised learning to automatically establish surrogate safety measures (SSM) thresholds. Different traffic states and corresponding transitions are identified with the three-phase traffic theory using high-resolution trajectory data. Meanwhile, the SSMs are mapped to the corresponding traffic states from the perspectives of time, space, and deceleration. Three models, including k-means, GMM, and Mclust, are investigated and compared to optimize the identification of traffic conflicts. It is observed that Mclust outperforms the others based on the evaluation metrics. According to the results, there is a variation in the distribution of traffic conflicts among different traffic states, wide moving jam (phase J) has the highest conflict risk, followed by synchronous flow (phase S), and free flow (phase F). Meanwhile, the thresholds of traffic conflicts cannot be fully represented by the same value through different traffic states. It reveals that the heterogeneity of thresholds is exhibited across traffic state transitions, which justifies the necessity of dynamic thresholds for traffic conflict analysis.

## Introduction

Understanding the dynamics of traffic safety and traffic flow is essential for developing interventions that can reduce the occurrence of vehicle conflicts. These conflicts, often precursors to actual crashes, provide valuable insights into the conditions that may lead to crashes. However, the existing body of research has largely relied on historical crash data for safety evaluation, which has inherent limitations such as inaccurate data, subjective interpretations, and inadequate risk mitigation strategies^1^. Moreover, the complexity of driver behavior, a key factor in crashes, is often oversimplified or overlooked in prediction algorithms^2^. On the other hand, SSMs and microscopic traffic data have been proven to be appealing and widely used for analyzing traffic safety performance^3^. Hence, the goal of this study is to identify conflicts and link them to traffic flow characteristics using empirical trajectory data. To address these limitations, this study sets out with two primary objectives:

To explore the mechanism of traffic conflicts through the lens of macroscopic traffic states. This objective is predicated on the understanding that dynamic traffic states, with their spatiotemporal characteristics, serve as effective indicators for conflict detection. Macroscopic traffic flow characteristics have a profound impact on safety performance^4^, necessitating a deeper examination of how these characteristics correlate with the incidence of traffic conflicts. The transition between traffic states and the relationship of traffic parameters are critical to the causality of traffic conflicts. We aim to analyze traffic flow at various levels, such as Levels of Service (LOS), three-phase theory, and the fundamental diagram, to establish a connection between traffic flow parameters and conflict causality. This approach challenges the conventional reliance on micro-traffic flow features for conflict forecasts and aims to provide a more comprehensive understanding from a macro perspective^5^.

To utilize empirical trajectory data to identify traffic conflicts and determine Surrogate Safety Measures (SSMs) indicator thresholds. This objective seeks to transcend the limitations of previous studies by focusing on the mechanisms of conflict and the inherent heterogeneity in traffic flow, as revealed by high-resolution trajectory data. The selection of different thresholds of various scenarios can help us better understand the correlation between traffic conflicts and traffic flow parameters. The study intends to develop dynamic thresholds for traffic conflict analysis, which is particularly relevant for the algorithm development of automated vehicles (AVs), providing nuanced assessments of conflict severity in relation to traffic states^6^.

By fulfilling these objectives, this study aims to contribute to the field by enhancing the understanding of the relationship between traffic conflicts and traffic flow characteristics, leveraging high-quality trajectory data. This contribution is crucial, as it has the potential to inform the development of more accurate predictive models and safety interventions, ultimately leading to safer road environments.

The structure of the remaining research is outlined as follows: Section “Literature review” presents a comprehensive literature review. The methodology employed in this study is detailed in Section “Methods”. Section “Results” reports on the findings and discussions of the analysis. Finally, Section “Conclusions” concludes with the implications of the study’s findings and suggestions for future research endeavors.

## Literature review

### Traffic flow and states

The performance of traffic safety like crash occurrence is heavily influenced by traffic flow and their correlation has been studied by several studies. A link between traffic characteristics and daytime freeway crashes is established to confirm the importance of flow variation in traffic safety^7^. High-resolution trajectory data is applied to evaluate heterogeneous crash mechanisms under different traffic states^8^. However, crashes may not occur in many conditions, where safety evaluation is dependent on traffic flow characteristics and traffic conflicts. Probabilistic neural network (PNN) models were separately developed to identify and predict rear-end collisions in both congested flow and free flow scenarios, using loop detector data^9^. A logit model with random parameters and heterogeneity in means and variances was used to investigate the relationship between conflicts and traffic flow characteristics^10^. In previous research on traffic flow and states, a three-phase traffic flow theory is developed based on expressway data^11^. The three-phase traffic flow theory divided traffic states into three categories: free flow(F), synchronous flow(S), and wide moving jam(J). When in a free flow state, traffic flow is at a low density and high speed without disturbance of other vehicles. Furthermore, high flow and speed distinguish synchronous flow. Compared with free flow, the average speed is slower, and the density is higher. While in a wide moving jam flow, the flow and speed tend to be zero, and the density reaches a maximum. Transition processes exist for free flow and synchronous flow (F—S), free flow and wide moving jam (F—J), and synchronous flow and wide moving jam (S—J). Transitions between these three phases can all be first-order transitions. Among them, the transition from free flow to wide moving jam requires two steps. First, free flow transforms into synchronous flow, which then generates wide moving jams. Based on the three-phase theory, traffic states and variables can be chosen to assess the relationship between traffic flow and safety performance^12^. Traffic safety analysis with three-phase traffic flow theory is conducted with aggregated traffic flow data^13^.

### Traffic conflicts

To comprehensively examine traffic safety concerns that involve drivers, vehicles, and roads, safety surrogate measures (SSMs) have been widely used to measure different dimensions of conflicts^14^. The benefits and drawbacks of various SSMs are summarized^15^. The performance of SSMs by six indices to calibrate threshold with naturalistic driving data is assessed^16^. Fuzzy Surrogate Safety Metrics are presented to distinguish between safe and unsafe situations for rear-end collision^17^. Modified SSMs were used to capture the probability and severity of collisions based on simulation^18^. Traffic safety is assessed at signalized intersections by simulator validity from perspectives of traffic and safety parameters^19^. A series of machine learning algorithms and statistical learning techniques are generally applied to determine factors of conflict and predict the occurrence of conflict. Different network models such as CNN^20^, LSTM^21^, and DNN^22^ were proposed to detect and predict traffic conflicts from traffic variables and SSM. Based on statistical methods, conditional logistic regression^23^, stratified sampling^24^, and multiple logistic regression models^25^ were used to estimate conflict risk. In addition, the Peak Over Threshold (POT) approach^26^ and Multivariate Extreme Value models^27^ were also widely used to identify conflict frequency.

### Surrogate threshold values

Identifying traffic conflicts and determining thresholds are key for assessing safety performance. Typically, previous studies define thresholds with one value, disregarding their suitability for their studies. Even within the same context, multiple thresholds were proposed for analyzing conflicts. For example, the range of TTC thresholds varied widely from 0.5 s to 6.0s at signalized intersections for rear-end^28^. It is observed the same problem with PET thresholds as well^29^. Given the wide variation in the prescribed surrogate thresholds, some researchers have estimated the thresholds empirically. There are several major approaches for measuring conflict thresholds as shown in Table 1. While some studies determine the threshold of SSM based on real crash data, the selection of thresholds of various scenarios can be significantly different. This work addresses this research gap and uses high-resolution trajectory data to analyze three-phase traffic states by different traffic flow characteristics. The main objective of this work was to propose clustering methods, imbalanced data processing, and unsupervised learning evaluation on conflict identification and threshold selection for future research. Thus, these contributions can be applied in different types of locations at various traffic states. This can help us better understand the correlation between traffic conflicts and traffic flow parameters, which may be applied to investigate the differences and associations between microscopic conflict and macroscopic traffic flow.

**Table 1:** Methods to measure conflict thresholds.

Method	Description	Advantage	Disadvantage
Correlational approach	(1) Cumulative density function (CDF)^19^ (2) Extreme value models and the observed crashes^30^ (3) ROC (receiver operating characteristics) curves^31^	(1) Identify relationships between different variables related to conflict. (2) Well-suited for analyzing large datasets, collected from traffic sensors or during naturalistic driving studies.	(1) A correlational approach cannot establish causation of conflict and traffic flow. (2) An external parameter may influence parameter estimation. (3) The nuances of individual conflicts might not be captured.
Distribution-based approach	(1) Bimodal histogram method^32^ (2) Percentile method^33^ (3) Deviation method^34^	(1) Utilizes statistical distributions for a more objective definition of traffic conflicts. (2) Can be tailored to various traffic conditions and roadway types through modification of distribution parameters.	(1) Data quality greatly influences outcomes, and errors or biases can significantly affect results. (2) Conflict varies with distribution choice and parameter settings. (3) Inaccurate models or assumptions may result in over- or underestimating safety concerns.
Classification methods	(1) Discrete choice modelling^35^ (2) Machine learning^36^	(1) Ensures uniform application of analysis criteria across varied scenarios, promoting fairness and objectivity. (2) Utilizes historical data to anticipate possible conflict points, aiding in preemptive measures. (3) Manages several variables simultaneously, capturing the intricate mix of elements influencing traffic disputes. (4) Machine learning models benefit from ongoing data refreshes, refining their precision and adjusting to changing traffic trends and conduct.	(1) The accuracy of classification methods is heavily dependent on the quality and quantity of the data. (2) Traffic conditions are highly dynamic; models may not adapt quickly enough to sudden changes. (3) There can be cases where conflicts are either missed (false negatives) or normal situations are flagged as conflicts (false positives).
Time series analysis	(1) Clustering methods^37^ (2) Permutation entropy of kinematic indicators ^38^ (3) Vehicle kinematic indicators^39^	(1) Ability to predict future traffic conflict points and times using historical data. (2) Identification of daily, weekly, and seasonal variations in traffic patterns. (3) Capability to spot unusual traffic patterns that might signal impending conflicts.	(1) Time Series Analysis often assumes stationarity or linear relationships, potentially limiting applicability. (2) May not rapidly adjust to unpredictable events affecting traffic, like construction or crashes. (3) Focuses on prediction rather than explaining the causes of traffic conflicts.
Extreme value estimation	(1) Mean residual life plot^40^ (2) Threshold stability plot^41^	(1) Explain the heterogeneity in thresholds. (2) Statistical-related conflicts analysis	(1) Requires high-quality traffic conflict and crash data over a longer period (2) Sensitivity and specificity need to be improved

## Methods

**Figure 1:**
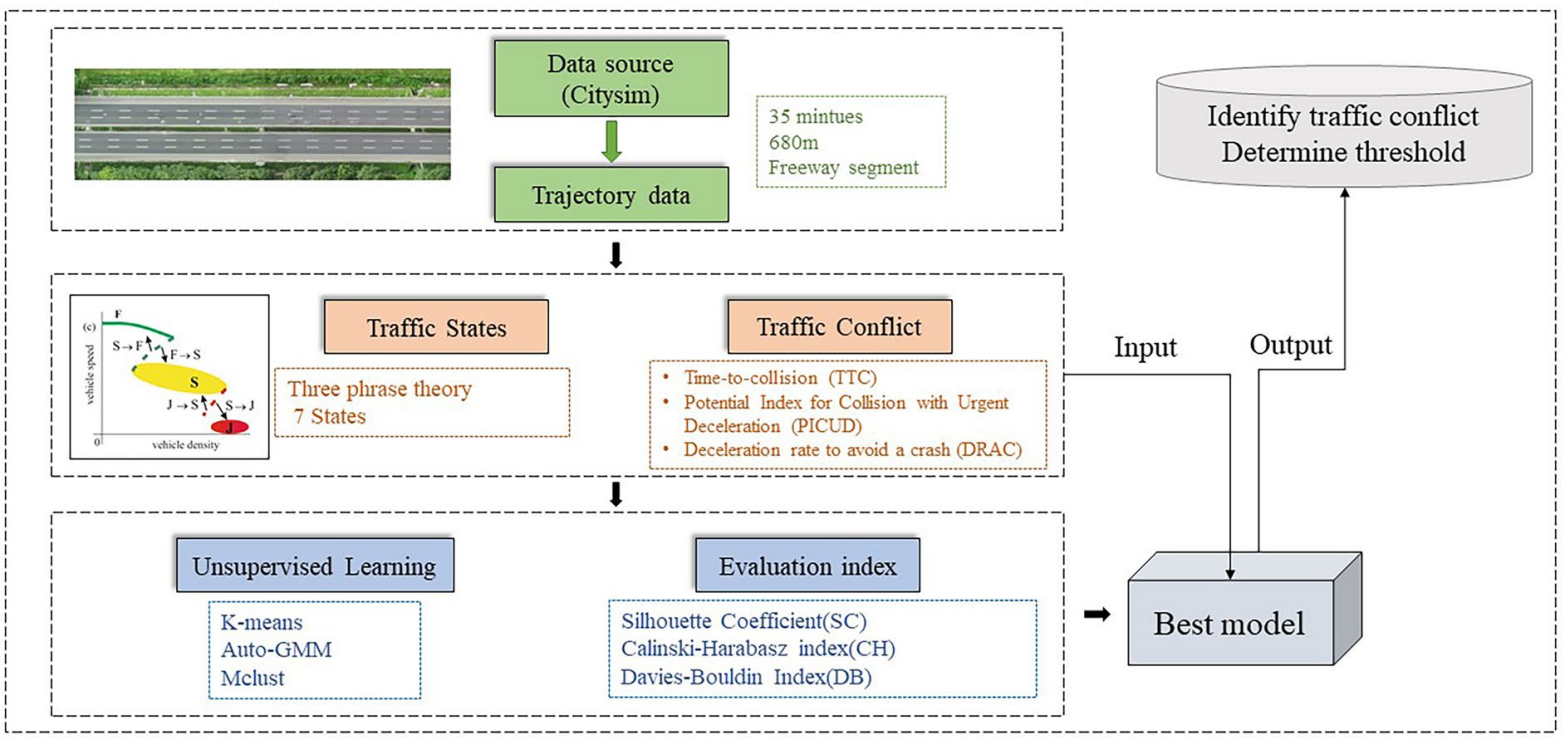
Diagram of the methodology.

The methodology of this paper is described in the following subsections: identification of traffic states, calculation of SSM, clustering methods, and evaluation. Initially, vehicle trajectory data is analyzed for freeway segments, and traffic flow variables such as flow rate, density, and average speed are calculated to classify traffic states according to the three-phase theory framework. Subsequently, the SSMs are computed for further study. Finally, unsupervised learning models including k-means, GMM, and Mclust are compared to automatically establish SSM thresholds. Figure 1 illustrates the proposed methods.

### Data preparation

**Figure 2:**
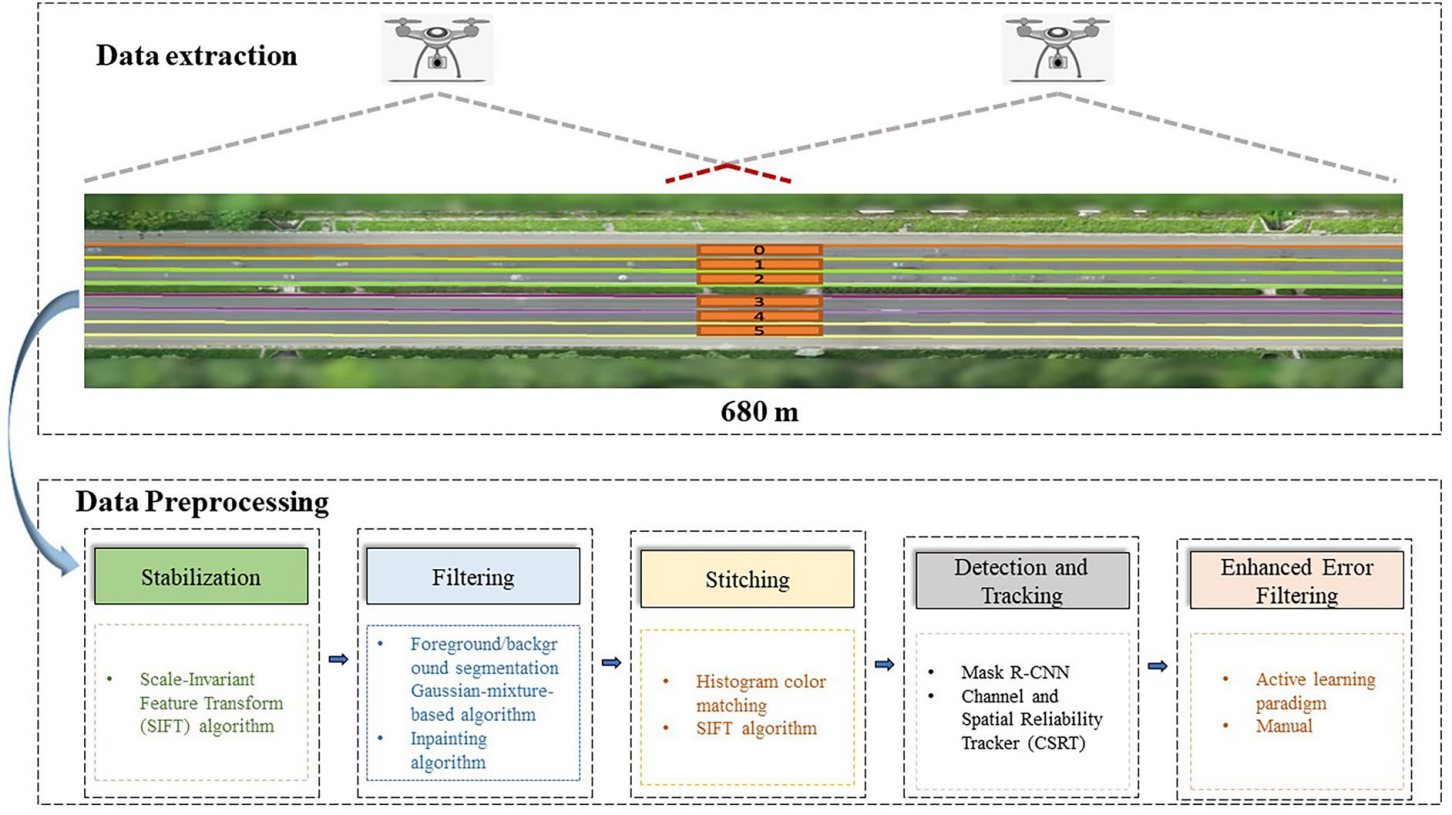
Aerial view of freeway segment and flow chart of data preprocessing.

This study is conducted with the “Citysim Dataset”^42^, an open-source dataset known for its remarkably high resolution of 4K (4096 × 2160) at 30 frames per second, captured from drone videos. Figure 2 illustrates a schematic diagram and an aerial view of the research area. The study area covers 680 m in length and consists of six lanes. During peak hours, a total of 35 minutes of data is available for the entire sample segment, divided into two periods: (1) 5:20 p.m. to 5:35 p.m., and (2) 5:48 p.m. to 6:07 p.m. To begin, vehicles are separated by lanes because traffic conditions differ between lanes.

**Figure 3:**
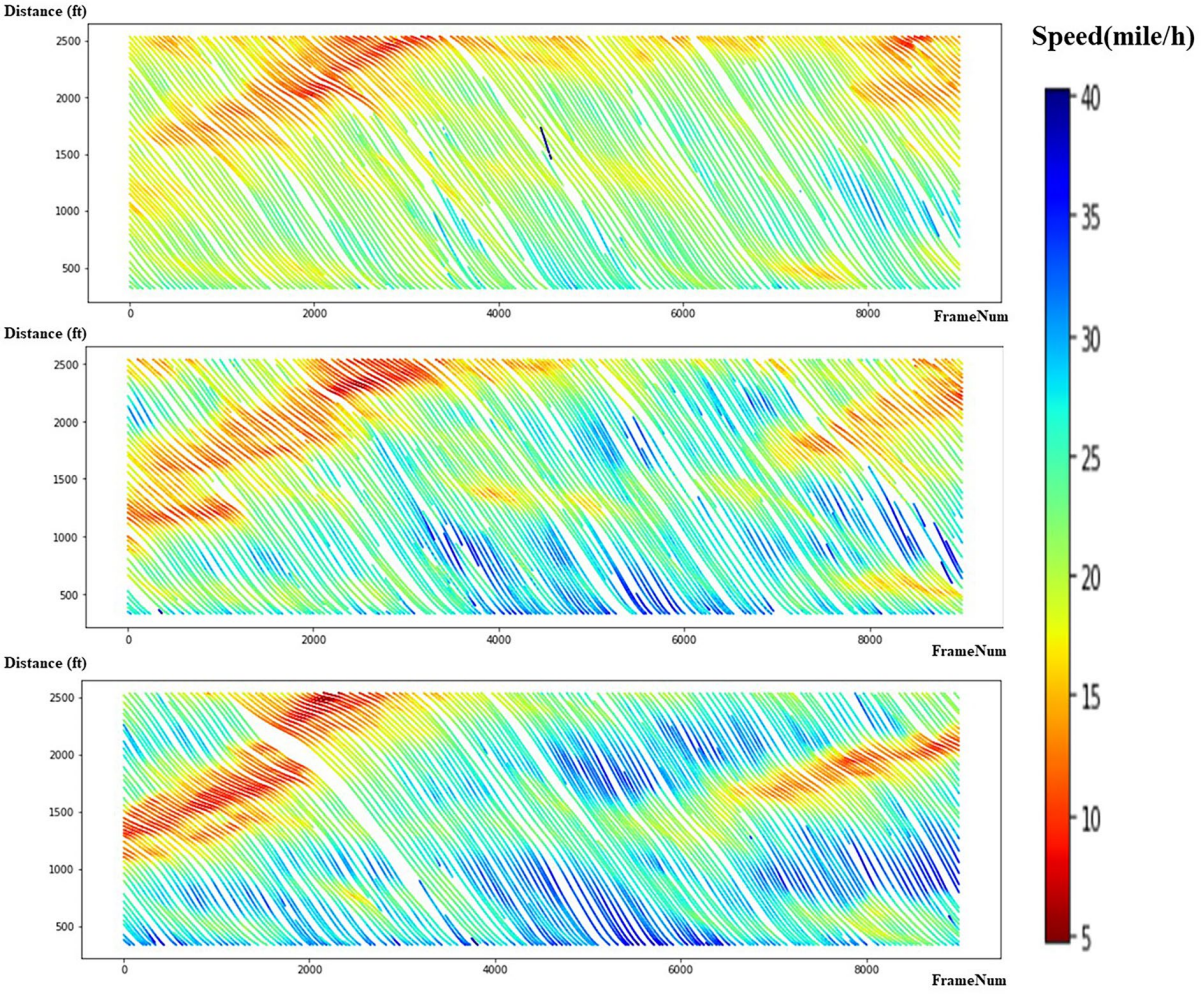
Vehicular trajectories with instantaneous speeds.

Following that, we filter out all lane-changing and cut-in behavior to focus on rear-end conflicts. To reduce noise, every 30 frames (1 second) are aggregated to quantify traffic characteristics and moving average method is applied to smooth the data. To streamline the process of locating the subject vehicle and its adjacent vehicles, the expressway has been segmented in both directions. Starting from each ramp and extending 100 meters in either direction, the expressway has been subdivided into 14 sub-segments. Vehicles in each sub-segment are paired to ensure that the trajectory remains continuous. Furthermore, the first and last vehicles in each video are removed. Following the preprocessing of data, many traffic flow characteristics are calculated to identify traffic states. We select space speed as one of the indices to evaluate macro traffic states, which is calculated by the average speed of all vehicles in the road segment. Density is defined as the number of vehicles divided by the length of the road segment. Furthermore, the flow rate is formulated by the average time headway: $q\left(flow \,rate\right)=1/\overline{h }(time \,headway)$. Figure 3 provides a visual representation of vehicular movement westbound across different frames or time intervals. The horizontal axis displays the sequence of frames, sourced from the video recording. The vertical axis measures the distance each vehicle covers. The form of these lines illustrates the vehicle's motion over time: a straight trajectory implies consistent speed, whereas a bending or oscillating one signifies speed fluctuations. The first plot (lane 0 in Fig. 2) shows a prominent cluster of low-speed trajectories towards the beginning (left side) of the chart. The second plot (lane 1 in Fig. 2) displays more diversity in speeds, with many trajectories hovering in the mid-speed range (greens and yellows). The third plot (lane 2 in Fig. 2) reveals a distinct area where vehicles slow down (red zone) on the left.

### Identification of traffic state

The traffic flow is categorized into three distinct phases under the three-phase traffic theory: free flow (F), synchronized flow (S), and wide-moving jam (J). Each of these phases can transition into another through specific mechanisms, such as a sudden increase in vehicle density (F to S), the dissipation of congestion (S to F), an increase in congestion leading to a jam (S to J), or the clearing of a jam (J to S). Free Flow (F) is characterized by high speeds and low vehicle density. Vehicles move freely without significant interactions with other vehicles. Synchronized Flow (S) is marked by medium densities and speeds. There is a synchronization in speed among vehicles, leading to closely spaced vehicles moving at similar speeds. Wide Moving Jam (J) involves high density and very low speeds, often leading to complete halts. It is characterized by the presence of "jams" that remain spatially fixed while moving through traffic.

**Figure 4:**
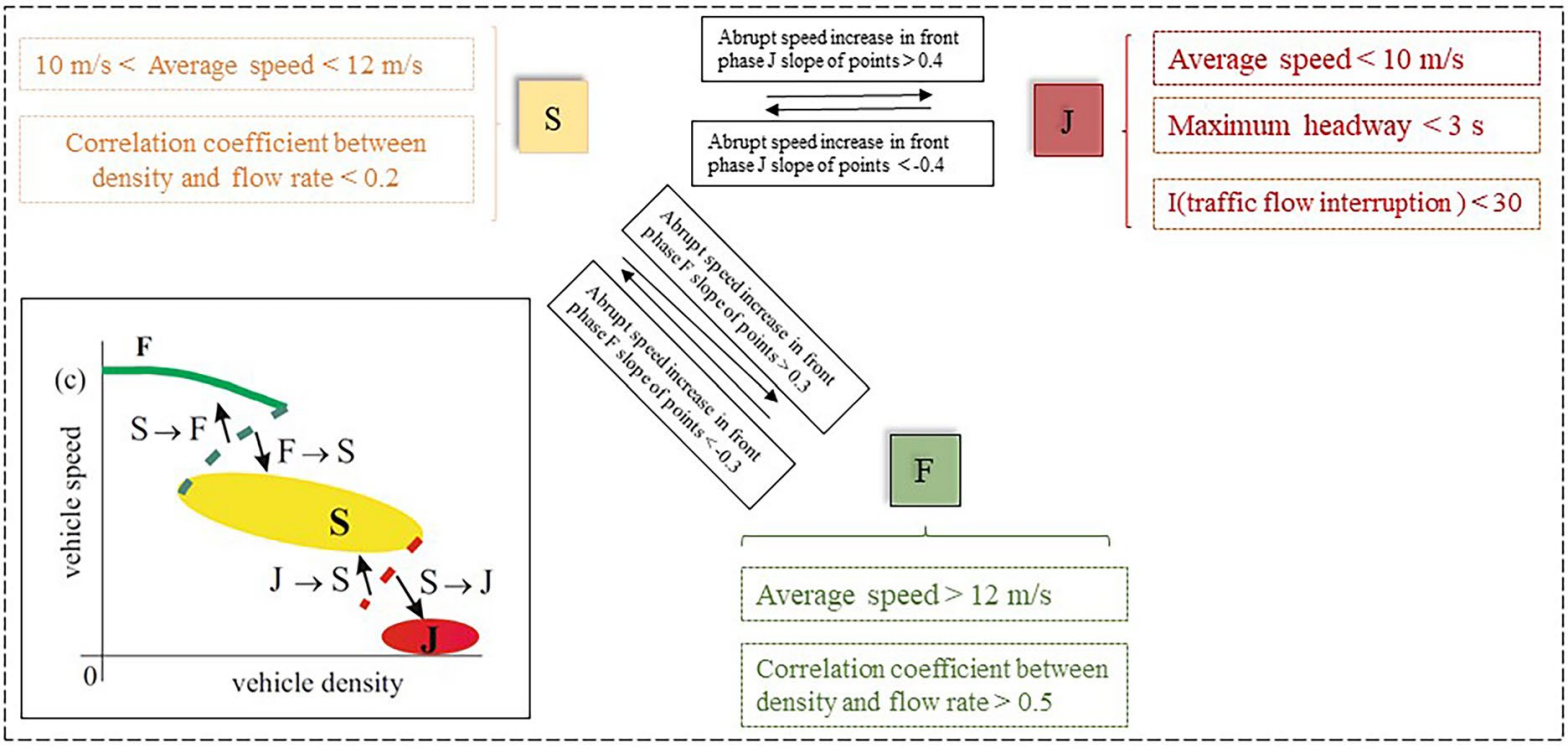
Process of traffic state identification.

The objective of this work is to connect conflict and traffic flow features, expanding the conventional conflict risk assessment to encompass the traffic flow condition. Figure 4 provides a visual representation of the criteria for traffic state identification. The traffic phase in a wide-moving jam can be determined by analyzing a time series plot that shows the effects of speed, time headway, and traffic flow interruptions. This analysis is conducted with several criteria, such as the average speed, maximum time headway, correlation coefficient between density and flow rate, and the number of vehicles in the phase.

The free-flow (F) phase is characterized by high speed (>12 m/s) and a strong connection between density and flow rate (> 0.5). In contrast, the correlation between density and flow rate in phase S is weak, with a correlation coefficient of less than 0.2. Meanwhile, the speed of Phase S was defined between 8 and 12 m/s. An abrupt change in speed characterizes the scenario that prevails between transitional phases F, S, and J^43^. There are three factors used to determine phase J. The low average speed (less than 8 m/s) was the first requirement. The maximum time headway (3s) was the second requirement. The third criterion involved a microcosmic interruption of the flow of traffic within a large moving jam^44^: (I<30 s), which is comparable to the quantity of vehicles in the jam. The calculation of I is shown in equation (1). Here, we set the I threshold as 30 to distinguish phase J.

A commonly used equation for calculating space speed (also known as space mean speed) in a stream of vehicles is given by Eq. 1:

$$\mathrm{Space \,\,Mean \,\,Speed }({\text{SMS}}) =\frac{\mathrm{Total\,\, Time \,\,Taken \,\,by \,\,All \,\,Vehicles }}{\mathrm{Total \,\,Distance \,\,Travelled\,\, by \,\,All \,\,Vehicles}}.$$

This formula can be further detailed as Eq. 2:

$$\mathrm{SMS }=\frac{{\sum }_{i=1}^{n}{d}_{i}}{{\sum }_{i=1}^{n}{t}_{i}}$$

where n = number of vehicles. ${d}_{i}$ = distance travelled by the ${i}{th}$ vehicle. ${t}_{i}$ = travel time of the ${i}{th}$ vehicle.

1 $$I=\frac{{\tau }_{J}}{{\tau }_{jam}},$$

where ${\uptau }_{{\text{J}}}$ is the duration of wide moving jam. ${\uptau }_{{\text{jam}}}$ is the the mean time in vehicle to pass the downstream of jam.

### Surrogate safety measures

**Figure 5:**
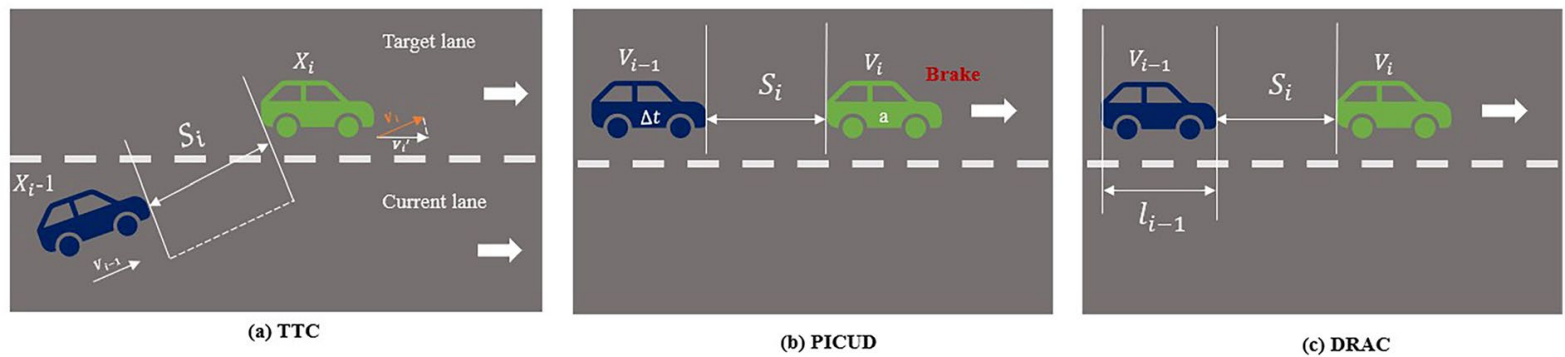
Illustration for the calculation of SSMs.

Traffic conflicts are used to predict interaction and the possibility of a crash if vehicles remain in their current state. Surrogate safety measures with a risk threshold can be used to assess conflicts^45^. It should be noted that there is no perfect conflict indicator for evaluating global conflict events. By classifying conflict indicators into three distinct types, a more profound comprehension of the interplay between conflicts and traffic flow can be attained. Rear-end collisions can be assessed using Time to Collision (TTC) as an appropriate temporal proximity indicator, providing insights into crash frequency and severity. To detect small crash probabilities and consider the road surface's friction coefficient in assessing pavement characteristics, the potential index for collision with urgent deceleration (PICUD) serves as a valuable spatial proximity indicator. Additionally, deceleration rate to avoid collision (DRAC), which combines a vehicle's maximum available deceleration rate, has been justified to be a reliable kinematic indicator for predicting rear-end crash risk. Figure 5a shows the speed and distance between two vehicles, which is crucial for calculating the TTC. The equation provided calculates TTC based on the distance between the vehicles and their speed difference. Figure 5b shows the calculation of PICUD, that leading vehicle braking and the following vehicle with a reaction gap, which is the distance that the following vehicle travels in the time it takes for the driver to react. Figure 5c illustrates the calculation of DRAC, which quantifies the deceleration requirement for the following vehicle to present a collision.  It considers the speed difference between the following vehicle and the leading one based on the distance between them excluding the length of the following vehicle. As a result, conflict measures must be chosen based on the research context. This research simplifies the computation process as well as identifies conflicts and their thresholds in a reliable manner. It should be noted that we chose the closest bounding box point between two vehicles rather than the center of the trajectory, which is more accurate to compute the SSM.

#### Time-to-collision (TTC)

Originally, TTC referred to the amount of time left before two vehicles would collide if they continued their current trajectory and maintained their speed difference. TTC is calculated using the equation (2).

2 $$TTC=\frac{{S}_{i}}{\overrightarrow{|{ V}_{i}}-\overrightarrow{{ V}_{i-1}}|}=\frac{{x}_{i-}{x}_{i-1}}{\overrightarrow{|{ V}_{i}}-\overrightarrow{{ V}_{i-1}}|},$$

where ${S}_{i}$ The distance between two vehicles, from rear bumper to front bumper. ${S}_{i}=$ ${x}_{i-}{x}_{i-1}$, $x$= the position of vehicles. ${{\text{V}}}_{{\text{i}}}$ The speed of a leading vehicle. ${{\text{V}}}_{{\text{i}}-1}$ The speed of a following vehicle.

#### Potential index for collision with urgent deceleration (PICUD)

PICUD is a measure that calculates the variation in distance between two consecutive vehicles in instances where the leading vehicle engages its emergency brakes. This calculation of PICUD is represented by assessing the change in the gap between the two vehicles during such emergency braking scenarios^46^.

3 $${\text{PICUD}}=\frac{{{{\text{V}}}_{{\text{i}}}}^{2}-{{{\text{V}}}_{{\text{i}}-1}}^{2}}{2{\text{a}}}+{S}_{i}-{{\text{V}}}_{{\text{i}}-1}\Delta {\text{t}},$$

where ${\text{a}}$ The urgent deceleration of a leading vehicle. $\Delta {\text{t}}$ The reaction time of a following vehicle.

#### Deceleration rate to avoid the crash (DRAC)

DRAC involves dividing the difference in speed between a following vehicle and a leading vehicle by the time interval between them. DRAC represents the rate at which the following vehicle needs to slow down to prevent a collision with the leading vehicle. The calculation is in equation (4):

4 $${\text{DRAC}}=\frac{{{({\text{V}}}_{{\text{i}}}-{{\text{V}}}_{{\text{i}}-1})}^{2}}{2({S}_{i}-{{\text{L}}}_{{\text{i}}-1})},$$

where ${{\text{L}}}_{{\text{i}}-1}$ The length of the following vehicle.

### Clustering methods and evaluation

In this section, we suggest evaluating the representation of SSM through unsupervised learning using three clustering models: k-means, GMM clustering, and Mclust. Since there are no ground-truth labels for traffic conflicts, internal evaluation methods and external evaluation methods are the two broad categories to evaluate clustering results. The external evaluation method assesses the quality of the clustering results while knowing the true label (ground truth), whereas the internal evaluation method does not rely on external information but only on the clustering results and sample attributes^47^. To assess the clustering outcomes, this research relies on internal metrics such as the Silhouette Coefficient, Calinski-Harabasz Score, and Davies-Bouldin Score.

A smaller ratio of Silhouette Coefficient Index indicates a greater distance between the sample point's cluster structure and the nearest cluster structure, which implies a better clustering result^48^. In addition, as the Calinski-Harabasz (CH) index decreases, the distance between clusters becomes smaller, suggesting a poorer quality of clustering^49^. The possible values of CH index range from 0 to infinity. Lastly, the range of the Davies-Bouldin Index is between [0, +∞). The clustering method performs good when the index is small^50^.fs

## Results

### Description of traffic state

The distribution of traffic flow variables at each traffic state is displayed in Table 2. To minimize interference, an output SSM sequence describing the interaction between each pair of vehicles was generated with a selected time step of one second (30 frames). To ensure that the simultaneity and variability of traffic states were accurately captured, the algorithm employed a sliding time-window analysis that allowed for the dynamic categorization of traffic states at any given moment. Classifying traffic states every 30 seconds. Summing the duration that each state was identified within the time windows for the entire study period.

**Table 2:** Description of traffic state.

Traffic state	Num of vehicles	Duration (s)	Flow rate (veh/h/ln)		Density (veh/m/ln)		Speed (m/s)		Time headway (s)	
			Mean	SD	Mean	SD	Mean	SD	Mean	SD
J	287	267	342.034	28.524	1.945	0.133	4.877	0.798	2.381	1.845
S→J	103	95	538.751	147.377	1.814	0.094	6.979	0.837	2.069	0.142
J→S	144	75	554.101	71.177	1.702	0.166	7.382	0.663	2.994	1.32
S	406	1113	624.002	52.478	1.47	0.034	9.536	0.444	2.396	0.61
F→S	109	60	491.809	11.186	1.424	0.093	9.308	0.332	3.735	0.057
S→F	78	90	511.203	49.092	1.33	0.08	9.714	1.234	3.626	1.085
F	147	339	482.644	5.987	1.038	0.045	12.9	0.143	3.097	0.067

The results show that phase S has the most cars over the longest period, followed by phases J, and then F. This phenomenon occurs because there is little abrupt turbulence throughout these three steady stages. Due to traffic congestion, phase J has the lowest speed and highest density, whereas phase F has the highest speed and lowest density among them. Phase S has the maximum flow rate owing to the large number of cars during this phase. Between phases J and F, phase S has a medium speed and density. In terms of transitional states, they do not last for long, and the levels of traffic flow variables were mild, compared to stable phases (F, S, J), which include fewer vehicles. These states have higher standard deviations than other states because they are undergoing unstable transitions and turbulence of traffic flow.

### Identification of traffic conflicts and thresholds

To validate the identification of traffic conflicts with different thresholds across various traffic states, matched traffic flow data of corresponding road segments is captured. The upstream refers to the traffic flow that occurs before a conflict point when considering the direction of traffic movement. To clarify, it starts when we first record information about each new vehicle that is spotted during a set period (which is 30 seconds for our study) as it enters the section of the road we're observing. Downstream refers to the traffic flow that occurs after the conflict point, again considering the direction of traffic. If we fail to locate the vehicle ID after the vehicle has left the road section at the downstream point, then the downstream location will keep a record of the last known details for that vehicle. The time of conflict is recorded by minTTC, which represents the smallest TTC value recorded among two distinct vehicle trajectories. The number of conflicts is represented by minTTC in our work. It signifies the most critical moment of potential collision between individual pairs of vehicles. Non-conflict observations are of utmost significance in studies as they showcase distinct characteristics that help discern conflict-prone situations by clustering methods. In this research, non-conflict observations refer to instances where no conflicts arise during the respective period and the subsequent timestamp (30s). Investigate the average values of SSMs for each identified cluster to establish threshold levels, which is shown in Table 3 with corresponding traffic index. For further study, it can be extended to delineate the perimeters separating clusters by examining the spread of SSMs for each grouping. Scrutinize the space spanned by multiple variables to ascertain the demarcation points for each one. These points typically occur where a cluster’s density begins to fade, potentially equidistant from the central points of adjacent clusters. Employing the clusters’ statistical characteristics, such as specific percentiles of SSMs within a cluster, can aid in setting benchmarks. These benchmarks can categorize varying degrees of traffic incident severity, ranging from high-risk to low-risk, and extending to non-conflict scenarios.

**Table 3:** Statistic summary of traffic conflicts and corresponding parameters.

Traffic state		All states with pre-set thresholds	All states	J	S→J	J→S	S	F→S	S→F	F
Threshold of TTC		1.500	3.572	2.982	3.414	3.540	3.446	3.578	3.444	3.989
Threshold of DRAC		3.400	3.493	3.493	3.425	3.692	3.356	3.460	3.503	3.814
Threshold of PICUD		0.000	0.052	0.041	0.043	0.045	0.064	0.051	0.049	0.081
Num of conflicts		162	297	67	51	32	38	25	14	18
Average upstream speed (m/s)	Mean	16.167	14.697	10.012	11.134	11.085	12.765	14.735	14.166	19.631
	Standard deviation	4.986	4.533	3.768	4.092	4.176	3.945	4.329	3.991	4.543
Average downstream speed (m/s)	Mean	16.077	14.615	9.895	11.352	12.129	11.478	13.994	14.263	19.894
	Standard deviation	5.480	4.982	2.566	4.864	3.978	4.189	5.646	4.564	5.897
Difference of speed between upstream and downstream (m/s)	Mean	3.024	2.749	2.145	2.356	2.800	2.012	2.532	2.128	3.523
	Standard deviation	2.063	1.875	1.163	1.843	1.496	1.238	1.391	1.472	3.329
Standard deviation of upstream speed	Mean	3.013	2.739	2.429	2.872	2.670	2.372	2.237	2.329	2.523
	Standard deviation	2.100	1.909	1.346	1.489	1.827	1.634	1.983	2.105	1.766
Standard deviation of downstream speed	Mean	2.937	2.670	2.297	2.989	2.526	2.234	2.303	2.145	2.496
	Standard deviation	1.337	1.216	1.223	1.380	1.842	1.961	1.721	1.841	1.236
Coefficient of variation of upstream speed	Mean	0.237	0.216	0.243	0.258	0.241	0.186	0.152	0.164	0.129
	Standard deviation	0.054	0.049	0.046	0.024	0.043	0.029	0.035	0.091	0.043
Coefficient of variation of downstream speed	Mean	0.245	0.222	0.232	0.263	0.175	0.208	0.260	0.150	0.125
	Standard deviation	0.085	0.077	0.056	0.097	0.065	0.086	0.064	0.043	0.079
Upstream traffic volume (Veh/30 s)	Mean	9.556	8.688	6.671	7.465	7.163	8.165	7.465	8.891	9.465
	Standard deviation	5.387	4.898	4.894	3.745	4.748	5.456	3.841	4.642	3.841
Downstream traffic volume (Veh/30 s)	Mean	9.684	8.803	6.784	7.164	7.984	8.065	7.135	9.165	9.723
	Standard deviation	5.630	5.118	4.215	4.489	4.921	4.984	4.413	4.654	4.895
Difference of volume between upstream and downstream (Veh/30 s)	Mean	− 0.127	− 0.116	− 0.113	0.301	− 0.821	0.100	0.330	− 0.274	− 0.258
	Standard deviation	1.281	1.164	3.256	0.310	1.416	0.465	0.894	0.413	0.654

Three models are compared from different theoretical perspectives to examine the performance of classification by unsupervised clustering. The input considers all traffic conflict variables, including PICUD, TTC, and DRAC. The Mclust model performs better than other clustering techniques, with results shown in Table 4.

**Table 4:** Clustering performance.

	K-means	GMM cluster	Mclust cluster
sc_index	0.65	0.675	0.72
ch_index	1063.78	1716.55	1836.95
db_index	0.86	0.91	0.63

**Figure 6:**
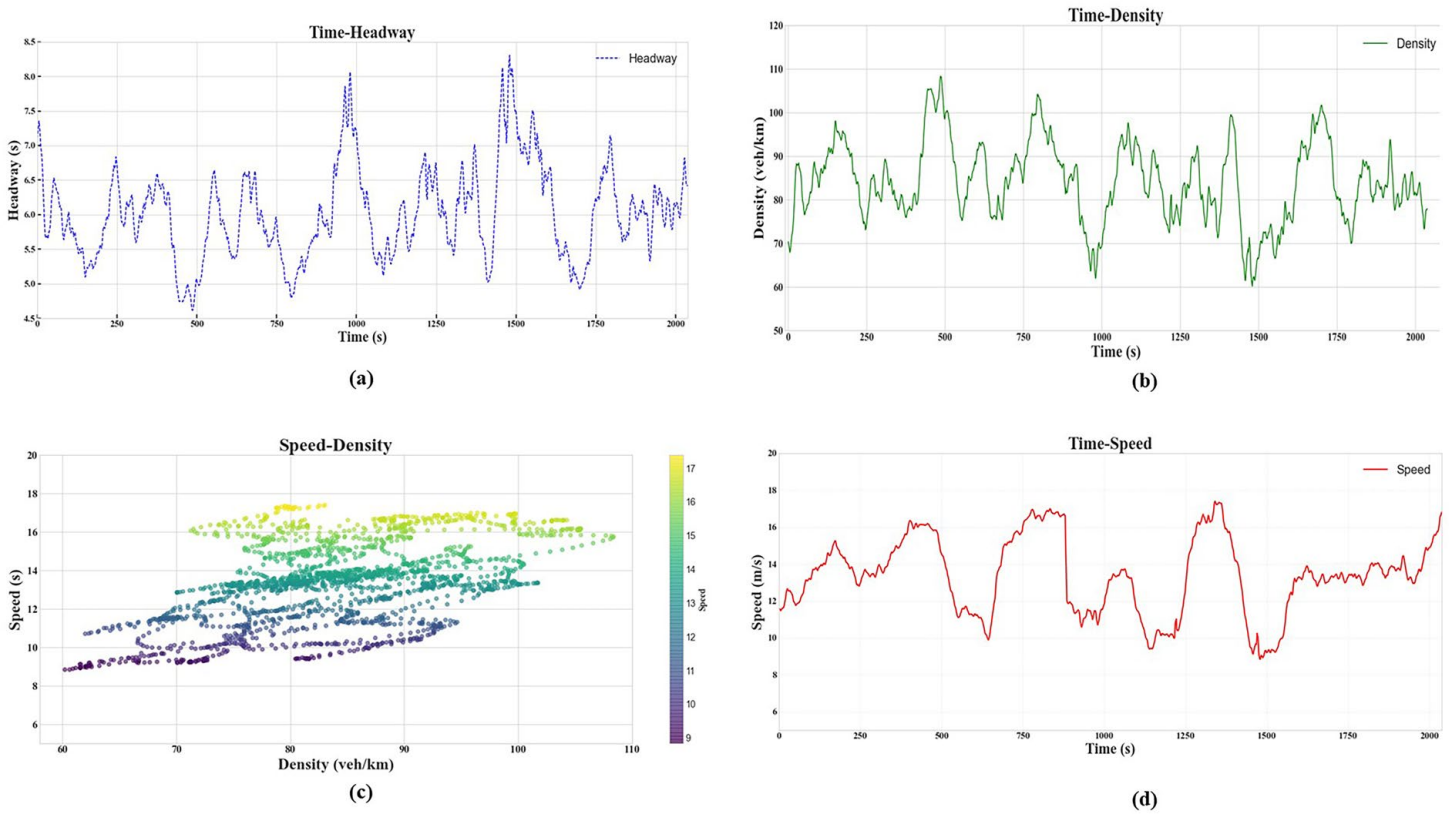
Macroscopic traffic conditions (**a**) Time- headway plot (**b**) Time- density plot, (**c**) Speed-density plot (**d**) Time- speed plot.

Compared with all states with pre-set thresholds, using the method proposed in our work can detect more conflicts. The thresholds of TTC and DRAC are higher than the common value in previous study since it considers the traffic condition of Freeway segment. When considering the specific traffic states, we can identify more details about traffic characteristics across their transition and link the relation between traffic conflicts and traffic flow characteristics (Fig. 6). Within Fig. 6a, the variation in headway—defined as the time gap between vehicles—presents a notable pattern for the observation period. Instances of decreased headway suggest a tightening of the gap between vehicles, which typically is associated with increased traffic density or a decrease in traffic speed, which is consistent with Figs. 6b and c. Such repeated occurrences of reduced headway might signal regular intervals of traffic congestion, whereas prolonged periods of expanded headway are indicative of less congested, free-flowing traffic conditions. Figure 6b reveals that greater vehicle density often leads to a reduction in speed, a trend that does not necessarily equate to a decrease in headway provided that the traffic flow remains consistent. This implies that even in dense traffic conditions, if the flow is steady and uninterrupted, vehicles may maintain a uniform headway. In the case of Fig. 6c, the inverse relationship between vehicle density and speed is depicted, with a scattered distribution of data points suggesting a variability in traffic behavior. At lower densities, vehicle speeds are high and diverse, pointing to a free-flow state. Conversely, as vehicle density rises, the spread of speed narrows, indicating a constrained flow and potential traffic congestion. This transition and narrowing of speed variation may reflect the onset of congested traffic conditions, characterized by reduced and more uniform vehicle speeds. The time-speed relationship (Fig. 6d) is characterized by its variability, with alternating peaks and valleys suggesting fluctuations that could be attributable to common traffic patterns, such as heavy congestion, or other transient influences on traffic velocity.

The thresholds vary significantly among these states. Phase J has the lowest threshold of TTC, and the traffic characteristics exhibit the same tendency of distribution. The average speeds and volumes of upstream and downstream are the smallest among all the states and exhibits lowest speed changes. The difference in volume between upstream and downstream is smaller than in other states, which indicates the lower variablity in traffic flow. The high coefficient of variation for speed is larger during this phase, which indicates that the speed is more spread out in relation to the mean, leading to higher variability and less homogeneity. Since phase F has the greatest threshold of TTC with the same tendency of average speed and flow, which allows drivers to have more time to respond to emerging systems, making it safer than other traffic states with fewer conflicts. The smallest coefficient of variation for speed suggests that speed has less dispersion and is more homogeneous during phase F. When a vehicle is in phase S, it keeps a similar deceleration. Due to large traffic volumes in phase S, vehicles in this phase have large thresholds, which means it requires more distance for a vehicle to avoid conflict. Other than stable phases, the transition between these phases has a larger threshold compared with phase J and S. This is because the vehicle tends not to maintain the same deceleration before a crash at these phases. The turbulence of deceleration will result in changes in the remaining distance between the leading and following vehicles. The larger standard deviation and coefficient of variation of average speed indicates a more extensive spread of the speeds of different states, reflecting greater variability across the transition of traffic states. DRAC aligns with our observations in TTC. Compared with the value (3.5/s^2) selected in most research, the threshold of DRAC is more accurate and sensitive to the changes in the flow and speed of vehicles. To be noticed, the threshold of PICUD is selected as 1 in all the states, which is a comparison of thinking distance and braking distance, expressed as a ratio or percentage.

Based on the PICUD, TTC, and DRAC values obtained from the Citysim Dataset, phase J poses the highest risk of conflict when traffic flow is extremely heavy and congested. Phase S follows with the second-highest conflict risk, which may be due to the high density and small space headway between surrounding vehicles. The maximum space headway between vehicles explains why phase F has fewer conflicts than S and J (Fig. 6). Moreover, due to the various traffic features, the transitional stages between these phases experience more conflicts than phase F. Hazardous situations may arise during the transitional state, as drivers tend to alter their behavior by decelerating in response to stop-and-go waves, which can exacerbate conflicts. The risk of conflict is higher during the S→J and F→S transitional states than in other transitional states. The high flow rate and vehicle speed during the F→S transitional state implies significantly more dangerous situation than phase F.

## Conclusions

In this study, the three-phase traffic theory is utilized to establish a link between macroscopic traffic flow states and microscopic traffic conflicts. By analyzing microscopic traffic trajectory data, an unsupervised clustering method is proposed in this research to detect traffic conflicts and establish the SSM thresholds based on the three-phase framework. Initially, traffic states and their transitions are identified using the three-phase theory and traffic characteristics. Conflicts in each state of traffic were then assessed using SSMs including TTC, DRAC, and PICUD. After comparing various clustering methods, the conflicts and thresholds were clustered using Mclust method. The study demonstrates that phase J poses the highest risk of conflicts based on the conflict outcomes, with phase S following closely due to its substantial sample size. On the other hand, phase F exhibits better performance than the other phases. The transitional states exhibit comparable levels of conflict risk, with the S→J and F→S transitions displaying more conflicts than the other transitions. These results suggest that the distribution of traffic conflict varies depending on the traffic state. Meanwhile, the thresholds of traffic conflicts cannot be fully represented by the same value through different traffic states.

Additionally, our method offers a novel approach to traffic conflict studies, which typically rely on predefined thresholds that may not reflect the complexity of traffic states, demonstrating the advantage of diverse thresholds over one. In terms of technology development, the advanced detection of conflicts using bounding boxes rather than centers of vehicles can be integrated into the development of autonomous vehicle (AV) safety systems. These systems would benefit from a more granular understanding of the vehicle’s surroundings, thereby enhancing the AV's ability to respond to potential hazards. By incorporating this method, traffic management systems can dynamically adjust their conflict detection mechanisms based on the prevailing traffic phase, thus enhancing the accuracy and timeliness of safety measures. This is particularly useful for intelligent transportation systems which can integrate these findings in real-time to improve traffic safety and flow. Our approach is also more generalizable and feasible in traffic safety applications since it can be employed to study crash causality without relying on actual crash data, which is particularly valuable when crash data is scarce or noisy. Further research with larger and more diverse datasets could focus on the following research directions. First, more motion features (e.g. converging/diverging trend of traffic flow, microscope vehicle movement behavior patterns) could be factored into the traffic state partitioning approach to help better reveal the hidden information during traffic propagation. Second, several more surrogate safety measure indices can be compared at the boundaries to extend the severity of traffic conflicts in the disaggregate real-time safety analysis.

## Data Availability

The datasets used during the current study are available on GitHub: https://github.com/UCF-SST-Lab/UCF-SST-CitySim1-Dataset.
